# Starving the Beast: Limiting Coenzyme A Biosynthesis to Prevent Disease and Transmission in Malaria

**DOI:** 10.3390/ijms241813915

**Published:** 2023-09-10

**Authors:** Brendan F. Riske, Shirley Luckhart, Michael A. Riehle

**Affiliations:** 1 Department of Entomology, University of Arizona, Tucson, AZ 85721, USA; brendanriske@arizona.edu; 2 Department of Entomology, Plant Pathology and Nematology, University of Idaho, Moscow, ID 83843, USA; sluckhart@uidaho.edu; 3 Department of Biological Sciences, University of Idaho, Moscow, ID 83843, USA

**Keywords:** pantothenate kinase, pantothenamide, pantazine, pantothenate, phosphopantetheine, *Anopheles*, *Plasmodium*, vitamin B5, malaria control

## Abstract

Malaria parasites must acquire all necessary nutrients from the vertebrate and mosquito hosts to successfully complete their life cycle. Failure to acquire these nutrients can limit or even block parasite development and presents a novel target for malaria control. One such essential nutrient is pantothenate, also known as vitamin B5, which the parasite cannot synthesize de novo and is required for the synthesis of coenzyme A (CoA) in the parasite. This review examines pantothenate and the CoA biosynthesis pathway in the human–mosquito–malaria parasite triad and explores possible approaches to leverage the CoA biosynthesis pathway to limit malaria parasite development in both human and mosquito hosts. This includes a discussion of sources for pantothenate for the mosquito, human, and parasite, examining the diverse strategies used by the parasite to acquire substrates for CoA synthesis across life stages and host resource pools and a discussion of drugs and alternative approaches being studied to disrupt CoA biosynthesis in the parasite. The latter includes antimalarial pantothenate analogs, known as pantothenamides, that have been developed to target this pathway during the human erythrocytic stages. In addition to these parasite-targeted drugs, we review studies of mosquito-targeted allosteric enzymatic regulators known as pantazines as an approach to limit pantothenate availability in the mosquito and subsequently deprive the parasite of this essential nutrient.

## 1. Introduction

Malaria remains one of the deadliest diseases affecting humanity and is responsible for nearly 250 million cases and over 600,000 deaths annually [1]. Current malaria control efforts rely on a combination of vector control strategies to disrupt transmission of the malaria parasite and antimalarial therapies for human infections. Vector control strategies, including insecticide-treated bed nets, larval source reduction, and the use of insecticides against both adult and larval mosquitoes, have been instrumental in reducing the overall burden of malaria worldwide. Human malaria infections are primarily treated with artemisinin combination therapies to clear the parasite from infected individuals [2,3,4]. Unfortunately, both of these complimentary strategies are rapidly becoming less effective as insecticide resistance in mosquito vectors becomes more widespread and the *Plasmodium* parasite develops resistance against front-line drugs [2,4]. Additionally, the invasion and establishment of *Anopheles stephensi* into sub-Saharan Africa is likely to significantly increase the disease burden on this continent [5,6,7]. This mosquito species is highly adapted to urban environments, posing unique challenges for environmental and chemical control strategies [8]. Thus, there is an urgent need for innovative malaria control strategies targeting both the parasite and mosquito vector as our current tools become less effective. Targeting essential nutrients necessary for parasite development in the human and mosquito hosts offers a novel approach to controlling malaria parasites during multiple stages of the parasite life cycle.

One promising nutritional target is the coenzyme A (CoA) biosynthesis pathway, which converts pantothenate, also known as vitamin B5, into CoA for central physiological processes of all three organisms in the transmission triangle. Importantly, pantothenate and CoA are essential for parasite development in both the human host and mosquito vector [9,10]. Specifically, depriving parasites of pantothenate and disrupting the CoA biosynthesis pathway have been explored as novel approaches to blocking malaria parasite development and transmission. In this work, we will review the CoA biosynthesis pathway in humans, mosquitoes, and *Plasmodium* parasites and how manipulation of host and parasite pathways can be targeted to limit *Plasmodium* development and transmission.

## 2. Coenzyme A

Coenzyme A is an essential cofactor in many cellular processes, including glucose oxidation, fatty acid synthesis, ketogenesis, amino acid metabolism, and protein acetylation, where CoA acts as a carrier of activated acetate [11]. In addition, CoA has several key roles, including allosteric regulation of metabolic enzymes, control of post-translational modification of a number of proteins, and acetylation of histones to impact gene expression [12,13]. Protein acetylation via CoA is involved in the regulation of mitosis, autophagy, and apoptosis, and the ratio of acetyl-CoA to CoA is a key signal for the cellular balance of catabolic and anabolic processes [14]. In total, CoA and its thioesters are estimated to be involved in at least 4% of all cellular reactions [14].

### Pantothenate and CoA Biosynthesis

**Figure 1:**
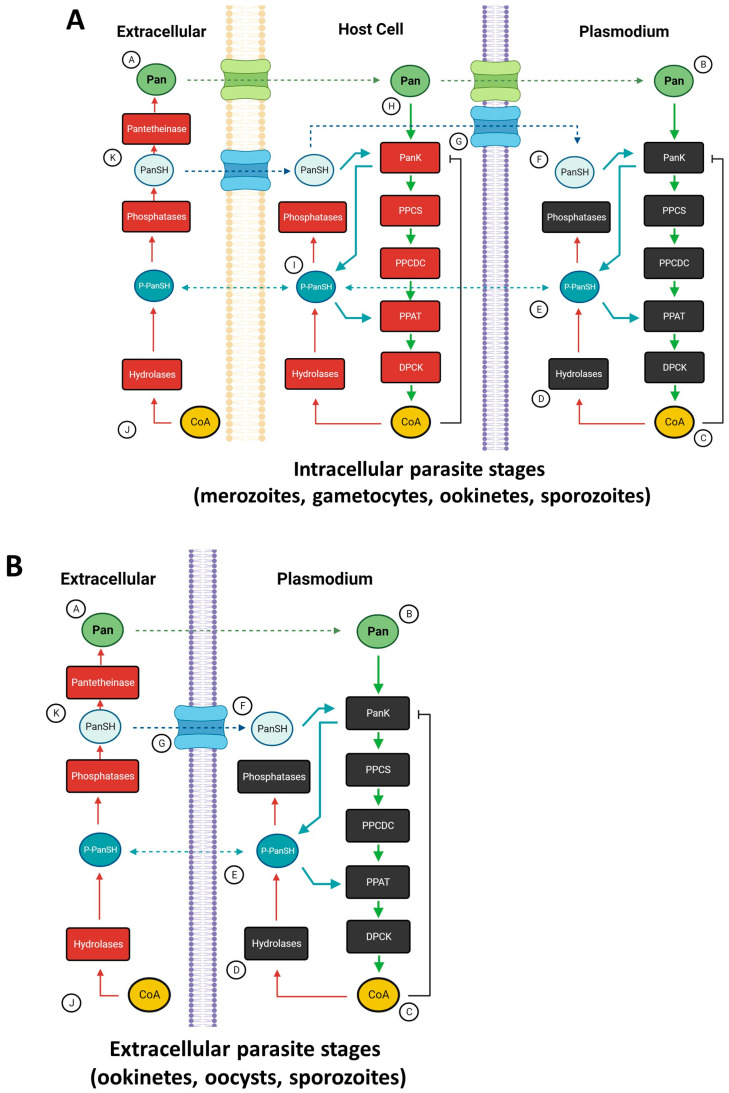
**CoA metabolism in the host and in *Plasmodium* spp.: Substrates, biosynthesis pathway, and regulation. ***Plasmodium* can acquire CoA precursors both within host cells (**A**) and extracellularly (**B**). During blood stage development, erythrocytes function as host cells, with plasma being the extracellular space. During the mosquito stage development, parasites are largely extracellular in the insect host, with intracellular transit during the ookinete and sporozoite stages. When parasites are intracellular, they utilize substrates from the extracellular space and the host cell for CoA biosynthesis, primarily via the canonical biosynthesis pathway, where pantothenate is imported into a host cell from the extracellular space (A). Pantothenate is transported by a parasite symporter, then enters the *Plasmodium* biosynthesis pathway (*Plasmodium* enzymes shown in black) via phosphorylation by pantothenate kinase (PanK) (B). Phosphopantothenate is subsequently modified by phosphopantothenoylcysteine synthetase (PPCS) and phosphopantothenoylcysteine decarboxylase (PPCDC) to generate phosphopantetheine (P-PanSH). P-PanSH is utilized by phosphopantetheine adenylyltransferase (PPAT) to generate dephospho-CoA, which is dephosphorylated by dephospho-CoA kinase (DPCK) to yield CoA. CoA negatively feeds back on PanK activity, which, together with substrate availability, are checks on CoA synthesis (C). CoA can be utilized for cellular processes or degraded into phosphopantetheine by endogenous hydrolases (D). Phosphopantetheine can enter the CoA synthesis pathway through PPAT, it can passively diffuse back into the host cell, or it can be degraded into pantetheine (PanSH) by endogenous phosphatases (E). PanSH can be converted to P-PanSH by PanK (F). PanSH is imported into host cells and *Plasmodium* by an unknown transporter protein (G). The host cell utilizes its own suite of CoA biosynthesis and recycling enzymes (host enzymes shown in red) (H). Importantly, P-PanSH produced by the host cell can passively diffuse into the parasite for use in CoA biosynthesis (I). Freely diffusing P-PanSH is available in the extracellular space, where degradation of pathway intermediates is enzymatically regulated (J). Specifically, CoA is converted by hydrolases into P-PanSH, which can then be converted by phosphatases to PanSH. PanSH can be converted into pantothenate in the extracellular space by pantetheinase (K).

CoA is predominantly synthesized via a 5-step enzymatic pathway initiated by the entry of pantothenate into the pathway. While there are other substrates and pathways for CoA synthesis, all intersect with the canonical CoA biosynthesis pathway (Figure 1) [15,16]. The first step in this pathway is the catalysis of phosphorylation of pantothenate (Pan) or pantetheine (PanSH) into phosphopantothenate (P-PanSH) by the rate-limiting enzyme pantothenate kinase (PanK) [17]. Phosphopantothenoylcysteine synthetase (PPCS) subsequently catalyzes the condensation of phosphopantothenate with a cysteine molecule to generate phosphopantothenoylcysteine. This is followed by the decarboxylation of the cysteine moiety by phosphopantothenoylcysteine decarboxylase (PPCDC) to produce phosphopantetheine. An adenylyl group is transferred to phosphopantetheine to yield dephospho-CoA, after which phosphopantetheine adenylyltransferase (PPAT) catalyzes the transfer of an adenylyl group to phosphopantetheine to synthesize dephospho-CoA. Dephospho-CoA kinase (DPCK) catalyzes the phosphorylation of dephospho-CoA to yield CoA [15,17]. In lieu of pantothenate, an alternative substrate, pantetheine, can enter the pathway following phosphorylation by PanK to phosphopantetheine, the substrate for PPAT [11,18,19]. Thus, both substrates for CoA synthesis enter the pathway at PanK catalysis. CoA and its thioesters negatively feedback to PanK, which helps to regulate intracellular levels of pantothenate and pantetheine [18]. CoA biosynthesis pathways are largely conserved among eukaryotes and bacteria, although considerable genetic and biochemical diversity among individual pathway enzymes exists [17,20,21,22].

## 3. Pantothenate

### 3.1. Pantothenate Synthesis

Pantothenate is required for most organisms to synthesize CoA. However, the capacity to synthesize pantothenate de novo is limited to various species of bacteria, fungi, and plants, and requires L-valine as an initial substrate, which is converted via three reactions to pantothenate [17,22]. Three enzymes, ketopantoate hydroxymethyl transferase (KPHMT), ketopantoate reductase (KPR), and pantoate-β-alanine ligase (PBAL, also called Pan synthetase), carry out this reaction. β-alanine is required for the final reaction. Organisms that lack this pathway, including humans, mosquitoes, and malaria parasites, must obtain the nutrient from primary producers or scavenge reserves from other organisms. Uptake from primary producers can be accomplished via diet or symbiotic relationships with pantothenate-producing microbes. Those organisms with narrow dietary ranges or parasitic lifestyles can find it challenging to obtain sufficient pantothenate for their survival [23,24].

### 3.2. Pantothenate Sources in the Malaria Parasite Life Cycle

**Figure 2:**
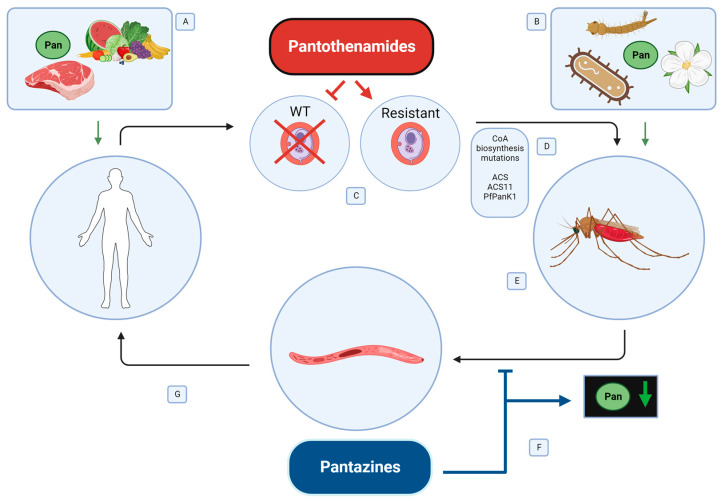
**Strategies for manipulating CoA biosynthesis to control malaria parasite transmission.** A combination of pantothenamide (red) and pantazine (blue) treatment could be used to disrupt malaria parasite development and mitigate resistance in human and mosquito hosts, respectively. *Plasmodium* must access pantothenate from humans and mosquitoes, which acquire the nutrient through diet and possibly bacterial symbionts (**A**,**B**). In human malaria, treatment with pantothenamides can disrupt the production of CoA in the wild-type (WT) parasite, leading to their destruction (**C**). However, this strong selective pressure can lead to the evolution of pantothenamide resistance in *P. falciparum* (denoted by Resistant) through mutations in CoA biosynthesis genes such as acetyl-CoA synthetase (*ACS),* acetyl-CoA synthetase 11 (*ACS11),* and PfPank1 (**D**). Resistant parasites can be transmitted to mosquitoes, but the resistance mutations lead to significant fitness effects. Once in the mosquito, both wild-type and pantothenamide-resistant parasites must acquire pantothenate from their mosquito host (**E**). Treating mosquitoes with pantazines that convert mosquito pantothenate into CoA can deprive mosquito-stage parasites of the pantothenate necessary for their survival (**F**). This could prevent the development of both wild-type and pantothenamide-resistant sporozoites, preventing transmission back to humans (**G**).

Humans obtain pantothenate through the consumption of plant and animal tissues (Figure 2) [15,16]. In the small intestine, pantothenate is absorbed by epithelial cells and passed into the circulatory system in a sodium-dependent manner [25]. Once in the circulatory system, pantothenate can be transported into cells for CoA synthesis. Dietary sources typically contain an excess of pantothenate necessary for cellular needs, making pantothenate deficiency relatively rare in humans who consume a typical diet of plant or animal tissues that are not pantothenate-limited [15]. Due to a typically high dietary intake, the gut microbiome is not considered a critical source of pantothenate for humans [16,26]. Further, while some gut bacteria synthesize pantothenate, it is unclear if humans access this supply and what, if any, role it plays in human health [27].

Like humans, mosquitoes primarily obtain pantothenate via their diet. Larval mosquitoes have the greatest access to pantothenate, as primary pantothenate producers make up a significant proportion of their diets. Mosquito larvae acquire food through filtering, suspension feeding, browsing, and interfacial feeding [28]. This allows them to ingest a variety of organic detritus, including bacteria and algae, many of which produce pantothenate de novo [17,28]. Thus, larvae are likely only pantothenate-limited when they develop in larval habitats with low food availability. In contrast, adult mosquitoes are primarily nectivores for which the nutritional quality varies greatly [29,30]. Nectar primarily consists of sugars, including glucose, fructose, and sucrose, along with low concentrations of amino acids, lipids, and vitamins [31]. The nutritional profiles of nectar can become modified by microbes and yeasts in the nectar, which can consume or add nutrients to nectar or be directly consumed by nectar feeders [32]. These nectar microbiomes can enhance the vitamin, protein, amino acid, or lipid content of nectar, which may also increase its attractiveness to nectivores [31]. It has been shown that many pollinators are sensitive to the nectar microbiome, including true fly pollinators in the order *Diptera* [31]. For example, the inoculation of flowers with the Gram-negative, non-motile bacterium *Acinetobacter nectaris* increased the number of visits by hoverflies, whereas the attractiveness of nectar of the tansy (*Tanacetum vulgare*) to *Culex pipiens* mosquitoes was enhanced by the presence of yeast *Lachancea thermotolerans*, which alters the odor profile [33,34]. While it has not been demonstrated that microbial communities can directly attract mosquitoes due to modification of the nectar nutritional content, sucrose supplementation studies suggest that mosquito fitness is improved by dietary consumption of nutrients found in microbially modified nectar. Experiments with male anopheline mosquitoes demonstrated that multivitamin supplementation enhanced longevity and fitness [35]. Similarly, female *Culex quinquefasciatus* provisioned with nectar supplemented with amino acids had increased longevity, while those provisioned with sucrose supplemented with vitamin B complex had significantly extended longevity and increased ovarian development [36]. Taken together, these studies suggest that mosquitoes feed on nectar both as a source of carbohydrates and a source of additional micronutrients.

While adult mosquitoes may obtain pantothenate from nectar, either in the nectar itself or from microbes in the nectar, adult consumption of pantothenate does not appear necessary for survival. Under laboratory conditions, adult mosquitoes are typically maintained on a diet of 10% sucrose, with no exogenous pantothenate. Adult female mosquitoes also consume blood, which provides the protein and lipids necessary for reproduction. While these bloodmeals are a source of pantothenate for the female, previous studies demonstrated that pantothenate levels in ingested blood are low, and it is unclear if the mosquito can acquire sufficient amounts of the nutrient from this source [23,37,38]. Blood feeding frequency is also highly variable and thus may not be a consistent source of pantothenate.

The limited availability of pantothenate in the adult mosquito diet suggests that pantothenate reserves are acquired during the immature stages. Larvae build reserves of lipids, carbohydrates, and proteins that persist into the adult stages, affecting key traits such as body size and fecundity [39,40,41]. In addition, larvae acquire non-energetic nutrients during this stage, including vitamins and salts [42]. Thus, larvae may store pantothenate and utilize this resource to improve adult fitness. Additionally, CoA generated during the immature stages can be recycled into pantothenate in adults if necessary [11,16]. The amount of pantothenate larvae acquire and how this impacts adult levels is an active area of study, although the heterogeneous nature of mosquito larval habitats, and thus pantothenate levels, make this challenging. Larval environments also play a key role in another possible strategy, the acquisition of symbiotic bacteria, for obtaining sufficient levels of pantothenate for survival.

### 3.3. The Mosquito Midgut Microbiome as a Source of Pantothenate

Many insects in addition to mosquitoes have diets limited in pantothenate. As noted above, this essential nutrient can be acquired through symbiotic relationships with microorganisms capable of synthesizing it de novo. This was first demonstrated in pea aphids (*Acyrthosiphon pisum*) that require *Buchnera* symbionts as a source of pantothenate [43,44]. Pea aphids feed on phloem sap, which lacks sufficient pantothenate for insect survival. *Buchnera* thrives in the pea aphid midgut and generates pantothenate, which can be absorbed and utilized by the host aphid. In another sap feeder, a more radical coevolution has enabled the insect host to acquire pantothenate that is unavailable in its diet [45]. In this case, the whitefly *(Bemisia tabaci*) midgut is colonized with the symbiotic bacterium *Candidatus Portiera aleyrodidarum* (hereafter *Portiera*) that has coevolved with these insects to provision them with pantothenate. Even more intriguing, *B. tabaci* has acquired a critical pantothenate synthesis gene by horizontal transfer from associated *Pseudomonas* bacteria [46]. Specifically, *B. tabaci* acquired *panB* and *panC* genes, which catalyze the conversion of oxoisovalerate to dehydropantoate and pantoate to pantothenate, respectively. The *panB* and *panC* genes are fused into a single gene with introns, known as *panBC*, which is expressed in whiteflies [45]. However, the *B. tabaci* genome lacks *ilvC*, which encodes an acetohydroxy acid isomeroreductase that converts dehydropantoate to pantoate, thereby preventing the direct synthesis of pantothenate by the whitefly. Instead, *Portiera*, which possesses an *ilvC* gene but lacks *panB* and *panC* genes, complements the insect host deficiency for de novo synthesis of pantothenate necessary for both organisms [45]. This symbiotic relationship is further enhanced by the presence of bacteriocytes, specialized tissues that harbor *Portiera* within *B. tabaci*, where *panBC* is expressed. The expression of panBC is negatively regulated by the *B. tabaci* microRNA (miRNA) novel-m0780-5p, which evolved after horizontal gene transfer occurred [47].

Mosquitoes possess several genera of associated microbes, but none have been identified as obligate symbionts [48,49]. Nevertheless, tissue microbiomes have been shown to impact the fitness of a variety of mosquito species [48]. The microbiome is primarily contained within the mosquito midgut, but the salivary glands, reproductive tissues, and hemolymph also harbor microbial communities, including bacteria in the genus *Wolbachia* that reside in cells of the ovaries or testes [49,50,51]. The midgut microbiome is dominated by Gram-negative bacteria, with species of *Pseudomonas, Aeromonas, Asaia, Comamonas, Elizabethkingia, Enterobacter, Klebsiella, Pantoea*, and *Serratia* being the most common [48,50].

The microbiomes of *Anopheles* spp. have been a major focus of research, as these microbial communities drive important interactions with malaria parasites [48,52]. The best-studied genera *Wolbachia* and *Asaia* form stable and persistent relationships with their anopheline mosquito hosts, although neither has been shown to play roles in B vitamin nutrition nor nutrition in general [53,54,55,56,57,58,59]. Despite this, whether other microbial associates in *Anopheles* spp. can synthesize B vitamins and, if so, whether their mosquito hosts can acquire sufficient quantities from this pool, are questions that will need to be addressed. Mosquitoes lack specialized bacteriocytes and, critically, no mosquito-associated bacteria have been identified as obligate symbionts, suggesting that diet, not the microbiome, is the primary source of pantothenate for mosquitoes [60].

### 3.4. Sources of Pantothenate for Plasmodium *spp.*

Like humans and mosquitoes, *Plasmodium* spp. do not produce pantothenate de novo, and thus these parasites must scavenge it from their mosquito or human host [61,62,63]. Such scavenging relies on a complex system to transport and store the nutrients (Figure 1). Pantothenate cannot passively diffuse into cells, so transporter proteins facilitate the uptake of pantothenate for use in the cytosolic synthesis of CoA or transport to membrane-bound organelles [19,25,63,64]. Mitochondria require significant amounts of CoA and have dedicated pools of pantothenate within their membranes for CoA synthesis [11,15]. While pantothenate transporter proteins are critical for the regulation of CoA synthesis, other metabolites assist in the homeostatic maintenance of pantothenate and CoA levels. For example, phosphopantetheine (P-PantSH) can passively diffuse across membranes and serve as an alternative substrate for CoA synthesis, bypassing the need for pantothenate transporters [65]. When P-PantSH is added to *Drosophila melanogaster* cells in vitro, it can passively diffuse across the cell membrane for CoA synthesis [65]. Because extracellular phosphopantetheine can be generated from the hydrolysis of CoA, extracellular CoA can be converted to intracellular CoA via P-PantSH as an intermediate. P-PantSH can also remain stable in serum for several hours and is considered a “nexus” metabolite, providing substrate for CoA synthesis across tissues in a membrane-independent manner [19,65]. P-PanSH likely plays a role in *Plasmodium* spp. CoA synthesis, at least during certain life stages. In the mouse parasite *Plasmodium yoelii,* only the last two genes in the canonical pathway, *PyPPAT* and *PyDPCK*, are essential for parasite growth during its blood stage, implying that only P-PanSH or dephospho-CoA are required by this parasite [62]. During mosquito stage development, all *P. yoelii* genes in the canonical CoA synthesis pathway are required, suggesting that the parasite must directly access pantothenate or pantetheine from the mosquito host [10]. The bird parasite *Plasmodium lophurae* can synthesize CoA from scavenged metabolic intermediates, specifically dephospho-CoA and P-PanSH [9,62,66]. Therefore, passive diffusion of P-PanSH could provide the necessary substrate for CoA synthesis in other *Plasmodium* spp., particularly during erythrocytic stages.

Pantetheine is another key intermediate that can be converted back into pantothenate or phosphorylated to enter the CoA biosynthesis pathway, bypassing the need for pantothenate [16]. Pantetheine can be generated during the recycling of CoA, when CoA is converted into P-PanSH and then to pantetheine by ectonucleotide pyrophosphatase/phosphodiesterase catalytic activity [16,65]. Extracellular pantetheine is unstable, however, because it is rapidly degraded into pantothenate by pantetheineases, which cleave a specific single amine bond in pantetheine, generating cysteine and pantothenate [16]. If pantetheine is taken up by neighboring cells and escapes this fate, it can be phosphorylated by pantothenate kinase to generate P-PanSH, which can then be transported through passive diffusion to where it is needed for CoA synthesis [19].

Taken together, these biochemical reactions provide two substrates for malaria parasite CoA biosynthesis. The first—pantothenate—can be transported from extracellular sources into the parasite for CoA biosynthesis. The second—freely diffusing P-PanSH—is available both intracellularly and extracellularly and can function as a substrate for CoA biosynthesis. *Plasmodium* spp. can leverage this flexibility to actively transport pantothenate from the extracellular environment or it can access passively diffusing P-PanSH from neighboring host cells and the extracellular space. P-PanSH can then be locked into the *Plasmodium* parasite by conversion to CoA, pantothenate, or pantetheine, none of which can diffuse out of the parasitized cell. By combining these strategies *Plasmodium* spp. can access a stable supply of substrate for CoA synthesis from its hosts.

### 3.5. Pantothenate Transport into Plasmodium Parasites

Since *Plasmodium* cannot directly transport CoA from the host, it must rely on obtaining the substrates described above, primarily pantothenate [17,19,61,65]. During the blood stage in the human host, *Plasmodium* parasites induce new permeability pathways (NPPs) in the host cell membrane. These NPPs increase erythrocyte permeability to a range where low molecular weight molecules, including amino acids, monosaccharides, ions, and vitamins, including pantothenate, can pass into the parasitized erythrocyte [63]. Once pantothenate is available in the erythrocyte, transporter proteins on the parasite cell membrane can acquire pantothenate [63]. Pantothenate is imported into the parasite using a low-affinity *Plasmodium* H+ symporter, a mechanism distinct from that of the Na+ transporters in mammals [16].

Putative pantothenate transporters (PAT) have been identified in several *Plasmodium* spp., with *P. falciparum* PAT (PfPAT) being the first to be characterized [25]. *PfPAT* encodes a parasite plasma membrane protein with sufficient conservation to complement the fen2 strain of Saccharomyces cerevisiae, which is pantothenate transport-deficient [25]. PfPAT was shown to be essential for erythrocytic development of *P. falciparum*, but *P. yoelii* PAT (PyPAT) was not required to form asexual or sexual stage parasites in the mouse host. However, PyPAT was necessary for oocyst development in the mosquito host [67]. Similarly, in *Plasmodium berghei*, knockout of a putative PAT gene did not affect blood-stage development, but *P. berghei* gametes were largely unable to escape host erythrocytes, so PAT-deficient *P. berghei* did not infect mosquitoes [64,68]. *P. berghei* PAT also plays a role in the excretion of key surface proteins during gametocyte and sporozoite stages. One possible explanation for these surprising findings is that blood-stage parasites of some species may have the capacity to scavenge CoA intermediates like P-PanSH and dephospho-CoA from its host cell, precluding the need for de novo CoA synthesis as has been shown for *P. lophurae* [9,69]. These findings and our own data suggest, however, that pantothenate uptake is perhaps the only mechanism whereby mosquito-stage parasites can acquire sufficient nutrients for CoA biosynthesis [10,38,70,71].

During mosquito-stage development, *Plasmodium* parasites have access to distinct nutritional pools in the insect host. Following feeding by the female mosquito, the ingested blood meal is surrounded by a peritrophic matrix (PM) within the midgut, a semi-permeable matrix of chitin and glycoproteins that facilitates digestion and temporarily restricts the parasite in the midgut [72,73]. During the first minutes to hours of digestion, parasite gametocytes are released from erythrocytes and mature into female and male gametes that undergo fertilization, with the resulting diploid zygotes becoming mobile ookinetes within the blood mass [73]. During this developmental period, the parasite could acquire pantothenate from the blood meal or it could rely on pantothenate or CoA stores acquired during erythrocytic development. Once the motile ookinete escapes the PM and traverses the midgut epithelium, it has access to pantothenate from mosquito midgut epithelial cells. After midgut invasion, ookinetes transform into sessile oocysts on the outside of the midgut, growing and dividing to generate hundreds of haploid sporozoites per oocyst, a lengthy and metabolically active period that likely utilizes a significant amount of CoA. Extracellular pantothenate in the mosquito hemolymph could serve as a pantothenate source for *Plasmodium* oocyst growth and development.

Manipulation of pantothenate levels in *A. stephensi* has affirmed that *P. falciparum* and *P. yoelii* oocyst development is dependent on pantothenate from the mosquito host. Transgenic *A. stephensi* with reduced pantothenate levels in the midgut exhibited reduced *P. falciparum* infection prevalence (percent of mosquitoes infected) and reduced mean midgut oocysts [71]. The provisioning of small-molecule PanK activators (pantazines) to *A. stephensi* similarly reduced midgut pantothenate levels and was associated with reduced *P. falciparum* infection prevalence and reduced *P. yoelii* infection intensity [10]. Effects on both species of parasites were reversed in the context of provisioning of a PanK inhibitor to *A. stephensi*. Since oocyst formation was inhibited by reduced pantothenate levels, nutrient-deprived parasites did not escape the midgut, ookinetes were unable to form viable oocysts, or the parasites perished during early oocyst formation. Following release from the oocysts, sporozoites transit through the host hemolymph, an environment with available CoA and related products, eventually reaching the salivary glands. Sporozoites may passively acquire phosphopantetheine, actively acquire pantothenate through membrane transport, or rely on pantothenate stores acquired during oocyst formation to generate CoA. The pantothenate requirements of sporozoites are unknown since CoA synthesis-impaired parasites do not reach this stage [67,73,74].

In summary, much of the evidence detailing the pantothenate requirements of malaria parasites comes from studies of the *Plasmodium* CoA synthesis genes, which are dispensable during some life stages and indispensable at others, depending on the *Plasmodium* species [17]. This has prompted detailed studies of parasite PanK genes and proteins, with an eye toward developing anti-malarial drugs.

## 4. Pantothenate Kinase

### 4.1. Structure, Function, and Expression of Pantothenate Kinases

PanK is the initial rate-limiting enzyme in the CoA biosynthesis pathway. Types I through III PanKs differ in their primary and tertiary structures, which define their biochemical characteristics. All three classes are found in bacteria, with most utilizing type I and a few possessing type II PanKs. Both type I and II PanKs are susceptible to feedback inhibition from CoA and its thioesters. Type III PanKs are found in only a few bacterial species and are not downregulated by increased CoA levels [17,20]. Eukaryotes, including *Plasmodium*, exclusively utilize type II PanKs, which will be the focus of this section [20,75].

The structure and function of type II PanKs, with domains A and B, are conserved across most eukaryotes [76]. The A domain possesses a pantothenate binding pocket and ATP binding site that facilitates the phosphorylation of pantothenate, while the B domain contains a dimerization interface and an allosteric inhibition site, which binds to both CoA and acetyl-CoA [76]. PanKs function as homodimers, with the two proteins connected via the B domain and the catalytic A domains on either side [76]. The inhibitory binding site spans the dimerization interface, and upon binding to CoA or acetyl-CoA, the dimer undergoes a conformational shift that prevents ATP binding to the catalytic pocket, which prevents pantothenate binding and inhibits PanK activity. The catalytic domain is conserved in type II PanKs, with unique N-terminal extensions in various organisms.

Type II PanKs have been studied in divergent eukaryotes, including humans, fruit flies, malaria parasites, and others, providing insights into the structure and function of these key enzymes. The human genome encodes three type II PanK genes, which encode four PanK isoforms that differ in tissue and subcellular localization [77,78]. PanKα and PanKβ are encoded by the *PanK1* gene with different start methionines for transcript expression [76]. PanKα is localized to the nucleus, while PanKβ is cytosolic. *PanK2* is exported to the mitochondria and nucleus, while *PanK3* is localized in the cytosol [76]. Arthropods studied to date, by contrast, utilize a single gene to produce multiple PanK isoforms. In *D. melanogaster*, *fumble* (*fbl*) encodes five PanK isoforms (fbl-RA through fbl-RE). These isoforms possess an identical catalytic domain but differ in their N-terminal extensions [78]. Fbl-RE (also known as fblL) has the longest N-terminal extension and is localized largely in the mitochondria, while fbl-RA (fblS1) and fbl-RB (fblS2) are cytosolic [78]. All currently available mosquito genomes encode a single PanK gene, but the abundance and function of potential isoforms have not been studied in detail. The *A. stephensi* PanK gene encodes at least two isoforms as verified by immunoblot and RNAi analysis [10]. One isoform has a long N-terminal extension and a considerably higher molecular weight than the other (67 kDa versus 42 kDa). As in the fruit fly, the long N-terminal extension of 67 kDa *A. stephensi* PanK might be consistent with mitochondrial localization in mosquito cells, although additional studies are needed to validate this.

Apicomplexans typically possess two PanK genes that encode two PanK isoforms, and *Plasmodium* spp., including *P. berghei, P. yoelii*, and *P. falciparum*, follow this pattern. In contrast to other eukaryotes where PanK is a homodimer, *P. falciparum* and *T. gondii* PanK1 and PanK2 can also form heterodimers. Heterodimer formation, however, is not essential as individual *Plasmodium* PanK isoform knockouts can be viable [66].

### 4.2. Pantothenate Kinase Disruption

The phenotypic effects of PanK deficiency or knockout vary in severity, from negligible when some cytosolic isoforms are disrupted to severe when mitochondrial isoforms are knocked out [78]. Disruption of the mitochondrially localized PanK2 isoform in humans results in the neurodegenerative disease Pantothenate Kinase-Associated Neurodegeneration (PKAN) [79]. Mutation of the PanK gene in *D. melanogaster* has provided insights into PKAN, with flies exhibiting severe motor dysfunction and the name *fumble* (*fbl*) as an allusion to this phenotype [80]. Hypomorphic *fbl*- mutants are also sterile and exhibit neurological degeneration and greatly reduced survival, with individuals rarely surviving past adult eclosion. Complementation of the *fbl*- background with human PanK2 and fbl isoforms demonstrated that PanK2 and Fbl-RE localized to the mitochondria, restored fbl functions, and reversed mutant phenotypes. Complementation with the human cytosolic isoforms PanK3 and PanK4 rescued most fbl- defects, except for male sterility [78]. This demonstrated that human PanK4 was a functional pantothenate kinase in vivo, which had been debated due to its lack of a conserved and proposed catalytic Glu/Asp residue [75,78]. It also provides evidence for the crucial functions of mitochondrial PanKs, as certain PanK deficiencies can only be rescued by their expression.

Apicomplexan PanK genes have been genetically manipulated to query function in several *Plasmodium* species [17]. In *P. falciparum*, *PfPanK1* is essential at every stage, whereas *PfPanK2* is not required [81]. This may be due to a transposon insertion in *PfPanK2* that inactivates protein catalytic activity [82]. While PfPanK2 can serve as a scaffold for heterodimerization of PfPanK1, it is not essential [82]. In *P. yoelii*, both PanK genes are individually dispensable during asexual development, with double knockouts being lethal [66]. However, knockout of either PanK significantly reduced oocyst formation during the sexual stages and largely blocked transmission during early development in the mosquito. *Plasmodium berghei* can survive the knockout of both PanK genes during the asexual erythrocytic stage, although the mechanism that allows for this survival is unknown [74]. Perhaps the parasite can scavenge CoA metabolites such as phosphopantetheine or dephospho-CoA to provision itself with CoA [62,65,66]. Knockout of either or both PanK genes does not affect ookinete formation, but as with *P. yoelii*, individual knockouts significantly reduced oocyst numbers [74]. Taken together, these observations suggest that some malaria parasites switch from utilizing phosphopantetheine for CoA synthesis during erythrocytic development to utilizing only pantothenate within the mosquito host. Since *P. falciparum* possesses only a single functional PanK gene and *PfPanK1* knockouts cannot complete asexual development, these parasites likely rely on PfPanK1 to phosphorylate pantetheine during both human and mosquito stages. The exclusive usage of pantothenate in mosquito hosts is further supported by our studies in which mosquito pantothenate levels were lowered indirectly through transgenesis or directly via small-molecule PanK activators as discussed above [10,38,70,71]. The essentiality of pantothenate for parasite sexual development makes mosquito hosts an ideal target to deprive *Plasmodium* parasites of this nutrient.

## 5. Development of Drugs Targeting the CoA Biosynthesis Pathway

### 5.1. Pantothenamides to Target Malaria Parasites in the Human Host

Since their discovery, *Plasmodium* PanKs have been an attractive drug target for novel drug development. Unfortunately, it has been difficult to target these enzymes due to the conservation of core catalytic domains in type II PanKs, which are used by many eukaryotes and bacteria. Compounds such as pantothenol and the pantothenamide CJ-15801 directly interfere with *Plasmodium* PanK and inhibit the growth of *Plasmodium vinckei* and *P. falciparum* in vitro and in vivo in a humanized mouse model [83]. This led to the development of additional pantothenate analogs, resulting in pantothenamides that exhibited activity at nanomolar concentrations under optimized in vitro conditions [17]. However, pantothenamides are susceptible to degradation in vivo by vanin family pantetheineases that cleave an amine bond in pantetheine to generate pantothenate and cysteine [84,85]. This amine bond is also present in pantothenamide drugs, which makes them susceptible to degradation [17].

To address pantothenamide instability, modifications were introduced to spatially alter the residues around the bond, including the addition of a methyl group to the distal amine bond [86,87]. The entire distal amine bond was displaced, in conjunction with the removal or addition of a methylene group, to create α-PanAMs and HoPanAms, respectively [88]. The amine bond has also been inverted to generate iPanAms [89,90]. Another strategy replaced the bond altogether with a triazole isostere, which cannot be degraded by pantetheineases [91]. These strategies produced several drug candidates, including N-PE-αMe-nPanAm, N5-trz-C1-Pan, and MMV693183, which exhibit anti-Plasmodium activity at nanomolar concentrations against the asexual blood stages of *P. falciparum*, *Plasmodium vivax*, and *Plasmodium knowlesi* in vitro [17,89,92]. Some compounds have exhibited activity against *P. falciparum* in a humanized mouse model, collectively revealing that novel pantothenamides are promising antimalarial drug candidates [17].

The mode of action of these pantothenamides has been a matter of considerable debate. The anti-Plasmodium effects have been attributed to competitive inhibition of pantothenate uptake, competitive inhibition of pantothenate phosphorylation by PanK, and inhibition of CoA-utilizing processes [17]. Recent evidence suggests CoA utilization is inhibited through the formation of antimetabolites [89]. Specifically, pantothenamides are activated by PanK phosphorylation and are further phosphorylated into analogs of other compounds in the pathway depending on the PanK enzyme targeted. Type I and type II PanKs can bind these compounds, while type III PanKs are resistant due to their high specificity for pantothenate [17]. In some organisms, pantothenamides remain bound to PanK, blocking the synthesis pathway. This is the likely mechanism of action of pantothenol, CJ-15801, and hopantenate against *Staphylococcus aureus* [93].

In *P. falciparum*, the novel pantothenamides appear to inhibit growth by the production of dead-end metabolites [94]. These compounds are generated as the pantothenamide moves through the CoA synthesis pathway in the parasite, eventually reaching a state where they cannot be utilized by the next enzyme in the pathway. The most commonly disrupted step is the formation of CoA-PanAms, which interferes with many downstream CoA steps [17]. Evidence for this comes indirectly from data showing that pantothenamide activity does not correlate with pantothenate phosphorylation or PanK activity [17,18,94,95]. Rather, CoA levels remain stable while acetyl-CoA levels are dramatically reduced, with support for this mode of action from mutations associated with parasite resistance against these compounds (Figure 2) [17]. In these studies, modification to an inverted amine bond pantothenamide (iPanAm), CXP18.6-006, resulted in the generation of pantothenamide-resistant *P. falciparum* parasites [94]. Four versions of this compound were tested, all with slightly modified side chains predicted to improve potency, and all four were converted into CoA-PanAms by the *Plasmodium* CoA biosynthesis pathway, which impaired parasite survival. Parasites were challenged with increasing concentrations of CXP18.6-052, which led to the development of resistant parasite lines [94]. All of these lines gained T627A mutations in the acetyl-CoA synthetase (*ACS*) gene, which altered an essential amino acid residue in the CoA binding pocket of the enzyme. Several lines also developed mutations in the related acetyl-CoA synthetase 11 (*ACS11*) gene, either E660K or K462N, also in the highly conserved CoA binding site. Engineering each of these mutations into susceptible *P. falciparum* using Crispr-Cas9 resulted in moderate drug resistance, which was increased with the insertion of two mutations [94]. In these resistant lines, CoA synthesis was detectable, but CoA levels were lowered by pantothenamide treatment implying that CoA production is inhibited in resistant lines by exposure to pantothenamides [94].

Resistance to pantothenol and CJ-15801 revealed additional functional mutations in *PfPanK*, including N507D, a deletion of an entire codon leading to a loss of glycine at residue 95 from *PfPanK1*, or a G-to-A substitution at that position [81]. These residues are adjacent to the PfPanK1 active site and are believed to alter enzyme stability or conformational changes. Engineering of these mutations into susceptible *P. falciparum* conferred pantothenamide resistance, resulting in significant impairment of the CoA biosynthesis pathway and reduced parasite growth. Resistant parasites, therefore, were less fit and were outcompeted by susceptible parasites in the absence of drug pressure [81]. Interestingly, these mutations resulted in resistance to some pantothenamides and increased susceptibility to others. PanOH-A-selected *P. falciparum*, with a substitution of N507D in *PfPanK1*, exhibited increased susceptibility to N5-trzC1-Pan, while the PanOH-B- and CJ-15801-selected lines exhibited increased resistance to this drug. PanOH-A-selected parasites were also more susceptible to N-PE-αMe-PanAm, while CJ-15801-selected parasites were more resistant, with PanOH-B showing no effect on these parasites [81]. It is likely that *PanK* mutations prevent pantothenamides from entering the biosynthetic pathway, limiting their conversion to unusable CoA-PanAms. However, some *PanK* mutations may not confer broad pantothenamide resistance if they can be phosphorylated by PanK. These data also revealed that pantothenol and CJ-15801 have modes of action that differ from N5-trzC1-Pan and N-PE-αMe-PanAm [81]. Thus, it should be possible to manage resistance with pantothenamides in combination or as part of a multi-drug strategy.

### 5.2. Pantazines to Target Malaria Parasites in the Mosquito Host

Pantothenamides target CoA synthesis in the malaria parasite, but there is increasing interest in the development of host-targeted strategies for malaria control [96,97]. For example, our data suggest that targeting the mosquito host to upregulate CoA biosynthesis and deprive *Plasmodium* of pantothenate can inhibit parasite development in the vector insect [10,38,71]. While this approach is not feasible in vertebrates due to potential side effects and their large stores of pantothenate acquired from their diet, the diet of adult mosquitoes is much more limiting as discussed above. Thus, this strategy leverages a new class of human PanK activator drugs called pantazines (PZ), which were originally developed as a therapy to treat PKAN in humans [98,99]. An early candidate, PZ-2891, activated PanK3 at nanomolar concentrations. A subsequent candidate, PZ-3022, was generated by replacing the isopropyl group on PZ-2891 with a cyclopropyl group, increasing drug resistance to degradation and its half-life in circulation [99]. Both PZ-2891 and PZ-3022 allosterically activate mammalian PanK3 to a similar degree but with slightly different pantothenate affinities [99]. Pantazines bind to the pantothenate site of the PANK3-ATP-Mg2+ complex, reorganizing the dimer interface [98,99]. CoA binding typically causes a conformational change, which prevents ATP binding to the complex, eliminating enzymatic activity. Pantazines induce a conformational change in homodimeric PanK, which obscures the CoA binding pocket and prevents CoA inhibition. The effects of pantazines are concentration-dependent [100]. At low concentrations, the majority of PanK protomers are not bound to pantazines, and thus PanK dimerization typically occurs between one bound protomer and one unbound protomer. In this case, the unbound protomer is held in an active configuration, rendering it insensitive to acetyl-CoA inhibition [100]. This results in increased PanK activity as one protomer is continuously active. In contrast, at higher concentrations, PZ-2891-bound protomers are likely to dimerize with other PZ-2891-bound protomers. This results in PanK dimers with no activity, as pantothenate has nowhere to bind when PZ-2891 is already bound to both protomers [101]. For PKAN treatment, a lower concentration of PZ-2891 is desirable as the goal is to stimulate PanK activity. In this context, CoA levels increase due to the constant activity of drug-bound PanKs. The observation that PZ-2891 binds to human PanK motifs that are conserved in other organisms suggested that pantazines might be functional in other species. For example, the *A. stephensi* PanK amino acid sequence is 80% similar and 65% identical to human PanK3. Crucially, *A. stephensi* PanK has identical residues that stabilize the allosteric dimer interface and interact with ATP, pantothenate, and acetyl-CoA in PanK3 [76]. Residues surrounding the PZ-2891 molecule show the isopropyl moiety enters the hydrophobic cavity of the pantothenate binding site, the carbonyl group hydrogen bonds to catalytic residue Arg193, and the pyridazine ring extends into the opposite protomer to form a hydrogen bond with Arg292′ and makes a p-p stacking interaction with Trp327′ [38,98]. These interactions are identical to the binding mode in PANK3 and predicted similar allosteric regulation of *A. stephensi* PanK regulation by PZ-2891 [10,101]. In our studies, we confirmed that provisioning of *A. stephensi* with PZ-2891 increased midgut CoA levels, presumably through the stimulation of PanK activity, while simultaneously reducing pantothenate levels [10,38]. The reduction in pantothenate stores in the mosquito host led to significant decreases in *P. falciparum* infection and decreased *P. yoelii* oocyst formation. Not only did these studies reveal that PZ-2891 could activate PanK in other organisms, but it showed that the drug was an effective anti-malarial when administered to mosquitoes [10].

## 6. Conclusions

Targeting the CoA biosynthesis pathway is an increasingly attractive strategy for combating malaria in both human and mosquito hosts, and promising pantothenamides have been developed. As with all new drug strategies, the potential for the development of resistance is a concern. The spread of resistant parasites occurs only through the mosquito host, so the potential to inhibit this spread through chemical or genetic enhancement of mosquito PanK activity could be leveraged for transmission blocking of both susceptible parasites and pantothenamide-resistant parasites. Utilizing a two-pronged strategy of PanK manipulation in both humans and mosquitoes will allow us to preserve the efficacy of pantothenamides and further reduce parasite transmission by the mosquito.

Delivering pantazines to mosquitoes under field conditions will require iterative field- and lab-based studies. Drugs could be delivered to mosquitoes via bait stations, where artificial nectar is spiked with pantazines [10,102,103]. Field trials of attractive toxic sugar bait stations for mosquito control are currently underway, with some promising results [103]. Nanomolar concentrations of PZ-2891 significantly reduced *A. stephensi* pantothenante [38], opening the possibility of identifying chemical modifications with greater efficacy at doses that could be imbibed cost-effectively from feeding at the station. The environmental stability of these drugs is untested, as is the impact of these drugs on off-target species. However, based on our observations that pantazine treatment had minimal to no effects on mosquito fitness [38], we are optimistic that shifting pantothenate stores to CoA in other arthropods would have similar minimal effects. In addition to deploying small-molecule PanK activators to mosquitoes in the field, engineering vector mosquitoes with enhanced endogenous PanK expression and reduced pantothenate levels could be an adjunctive strategy to pantazine delivery. In summary, CoA provides a novel and intriguing target for malaria control during all stages of the parasite development cycle. Targeting the parasite in both humans and mosquitoes provides multiple opportunities to block the infection, transmission, and spread of drug-resistant parasites.

## Data Availability

Data sharing not applicable.
